# Primary Amoebic Meningoencephalitis in an Iranian Infant

**DOI:** 10.1155/2012/782854

**Published:** 2012-07-26

**Authors:** Zahra Movahedi, Mohammad Reza Shokrollahi, Mohammad Aghaali, Hosein Heydari

**Affiliations:** ^1^Qom University of Medical Science, Qom, Iran; ^2^Department of Prdiatric Infectious Disease, Qom Children Hospital, Qom University of Medical Science, Imam Boulevard, Qom 37185-3458, Iran

## Abstract

*Introduction*. *Naegleria fowleri*, a free living amoeba, can cause devastating and deadly diseases in humans. This is the first report of primary amoebic meningoencephalitis from Iran. *Case report*. A five-month-old male infant presented with the history of fever and eye gaze for three days, after beginning of bacterial meningitis, a plain and contrast CT revealed communicated hydrocephalus. In the repeat of CSF analysis on microscopic examination of wet preparation of CSF, *Naegleria Fowleri* was seen. Then, Amphotericin B and Rifampin were started. On followup, two months later, the patient was totally asymptomatic. *Conclusion*. Though occurrence of PAM is rare, this unusual disease has grave prognosis, so infection with free living amoebas must be considered in differential diagnosis of pediatric patients of purulent meningitis without evidence of bacteria on Gram's stain and imaging findings, nonspecific brain edema on CT or hydrocephalus even without history of contact.

## 1. Introduction

Free living amoebae include many genera with high distribution in natural and artificial environment, such as air, water, dust, and soil. Among the many genera, members of only four genera have an association with human diseases: *Acanthamoeba spp., Balamuthia mandrillaris, Naegleria fowleri and Sappinia diploidea* [1].

*Naegleria fowleri*, a free living amoeba, can cause devastating and fatal diseases in humans [2]. It causes an acute and fulminating infection of the central nervous system, primary amebic meningoencephalitis (PAM), in healthy children and young adults who indulge in aquatic activities in fresh water. It was first described in 1965 by Fowler and Carter in Australia [3].

About 300 cases of PAM have been reported in the world, mostly in USA, Australia, and Europe. These infections are nearly uniformly fatal with only few survivors of PAM reported [4–9].

PAM is a fulminant infection that typically leads to death in first or second weeks from the onset of symptoms. The trophozoites, active stages of *N. fowleri*, infect the brain through the olfactory bulb by penetrating nasal epithelium [10].

Rapid progression of the disease process and limited awareness among the clinicians and diagnostic staff make the disease a diagnostic challenge.

Base of acknowledgment, till date, there have been no reports of PAM due to *N. fowleri* from Iran.

## 2. Case Report

A five-month-old male infant presented with the history of fever and eye gaze from 3 days ago. Fever was mild to moderate without any associated chills. 

His birth history as well as developmental history was uneventful. The child was immunized up to date. The mother had no signs of mastitis. The child was apparently asymptomatic until two days before admission.

There was no evidence of other focal deficit, altered sensorium, or seizures. Patient neither swam nor recently took a bath in pond, pool, or lake. All relevant history for tuberculosis was negative.

CBC showed WBC 20,500 /mm^3^ (PMN 65%, lymphocytes 30%, monocytes 1%, eosinophils 1%, band cell 3%), hemoglobin 10.5 g/dL, hematocrit 31.9%, platelet count 731,000 /mm^3^. Serum electrolyte showed Na = 138, K = 5.7. ESR and CRP were 92 mm/h and 11.3 mg/L, respectively. Stool exam and urine analysis were normal; blood, urine, and stool culture were negative.

 A plain and contrast CT revealed communicated hydrocephalus. Following CT, lumbar puncture was done and CSF sent for microbiological and cytological analysis which revealed milky color, 2500 WBC cells/mm^3^ (PMN = 75%, Lymph = 25%) without any RBC with 391 mg/dL protein and 3 mg/dL of sugar. No bacteria or fungal elements were seen on gram stain. Bacterial culture was negative. Provisional diagnosis of acute bacterial meningitis was made, and the child was treated empirically by intravenous ceftriaxone 100 mg/kg/day and vancomycin 15 mg/kg/dose. 

Smear and PCR of CSF for tuberculosis was negative. Four days after starting of vancomycin and ceftriaxone, fever has not decreased so CSF examination was repeated. Second CSF analysis revealed 150 WBC cells/mm^3^ (PMN = 70%, Lymph = 30%), RBC 500 cells/mm^3^ with 121 mg/dL protein and 16 mg/dL of sugar. *Naegleria Fowleri* was seen on microscopic examination of wet preparation of CSF.

Following the demonstration of amoebae in CSF, clinical diagnosis of primary amoebic meningoencephalitis (PAM) was made. Rifampin was started in a dose of 10 mg/kg orally per day and Amphotericin B in a dose of 1 mg/kg/day. CSF culture for Naegleria was also positive. 

**Figure 1:**
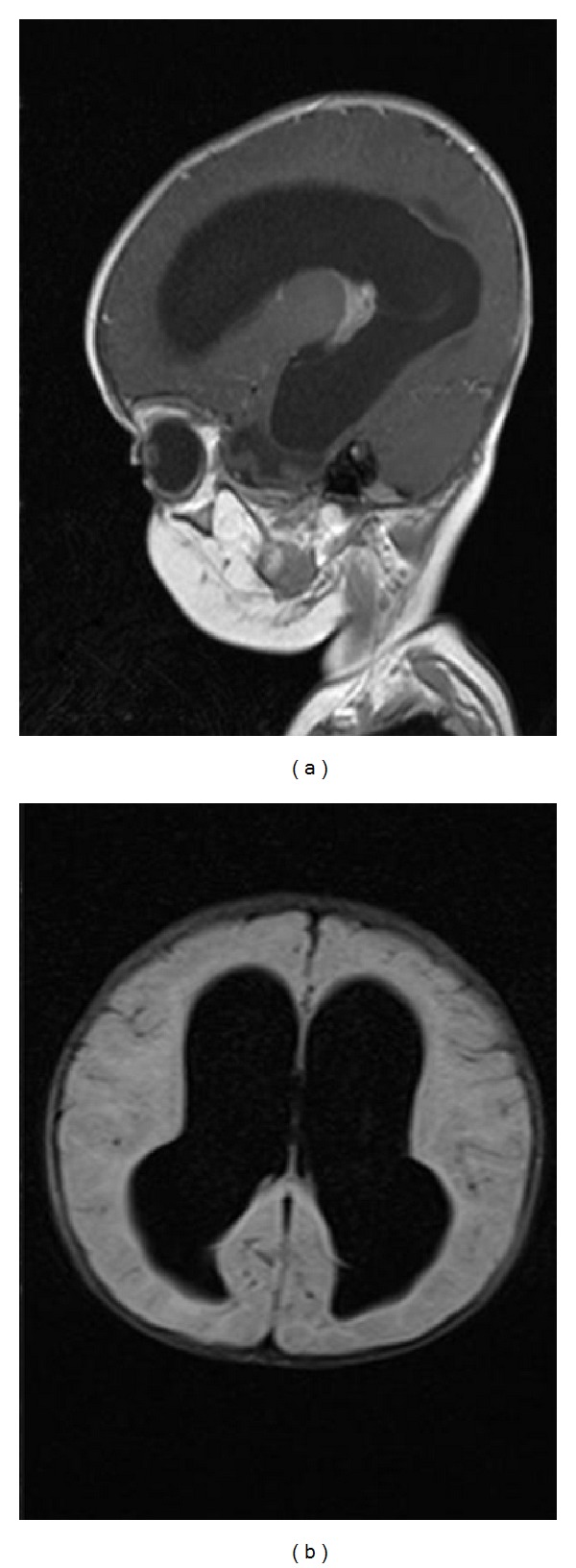
T1 sagittal (a) and T2 dark fluid transverse (b) view of brain MRI show hydrocephalus.

Three days after beginning of treatment with Amphotericin B and Rifampin, there was improvement in clinical signs and symptoms but fever has not decreased yet, so brain MRI was performed that revealed just hydrocephalus (Figure 1). The patient was referred to the Postgraduate Institute for the insertion of a ventriculoperitoneal shunt. Fever discontinued after surgery. After complete treatment, the patient was discharged. On followup, two months later the patient was totally asymptomatic.

## 3. Discussion

Primary amebic meningoencephalitis is divided into PAM and granulomatous amebic encephalitis. PAM is principally caused by *N. fowleri* in a patient with excellent health with prior intimate contact with fresh water, especially in summer. The portal of entry is via the olfactory mucosa and neuroepithelium. Incubation period is between 3 to 8 days with acute and rapidly fatal course. Patients usually die within 7–10 days of onset of symptoms. The clinical symptoms and signs of this patient were similar to those previously reported but hydrocephalus had not reported before. And this is the first report of PAM with communicating hydrocephalus. Most of the PAM cases are usually misdiagnosed as acute bacterial meningitis or tubercular meningitis [12, 16, 17]. In this case also patient primarily treated based on bacterial meningitis but he did not respond to treatment and in secondary evaluation PAM was diagnosed. 

Diagnostic test involves CSF study with direct visualization of Naegleria under light microscope which is actively motile and can be stained with Heidenhain's iron hematoxylin and Wheatley's trichrome stain [16]. To date, all pediatric patients with PAM diagnosed by wet smear (Table 1), in this report diagnoses have been done by wet smear, too; but unfortunately we do not have any image of CSF smear and culture.

Biochemical analysis of SCF can also help us to show pleocytosis with neutrophilic dominance and high protein with low sugar. In this case, the color of CSF was milky that has not reported in previous cases and pleocytosis with neutrophil dominance was observed. Indirect hemagglutination, ELISA, and indirect/direct immunofluorescence are other methods used for diagnosis. Although serology is not useful in diagnosis during the acute stage, as antibodies to Naegleria species have also been detected in a normal person [18, 19]. In brain CT of the patient with PAM, hydrocephalus, hemorrhagic edematous confluent foci, can increase intracranial pressure which might have been detected [15]. MRI is usually suggestive of cerebral edema with meningeal enhancement [20]. In this case, MRI revealed hydrocephalus (Figure 1).

Naegleria usually transmitted while swimming or diving in contaminated water and has also been reported from other sources such as tap water and air [21, 22], rare cases have been reported where the infection occurred either by inhalation of air borne cysts or by face washing [23]. Among 7 pediatric reports of PAM, only two patients have had the history of contact.

The drug of choice for treatment of PAM is Amphotericin-B. Rifampin or Tetracycline may be added for better results. All the survivors reported till dates were treated with Amphotericin B. 

## 4. Conclusion

 In conclusion, although PAM is rare and has poor prognosis, it should be considered in any patients of pyogenic meningitis without evidence of bacteria by staining, antigen detection and culture. CSF cytology of wet mount becomes mandatory in such cases as early treatment with amphotericin may improve survival.

There should be an increased awareness among the diagnostic staff for careful examination of CSF wet preparation for rapid diagnosis of Naegleria infection as early treatment may markedly improve the prognosis of disease.

## Figures and Tables

**Table 1:** Pediatric amoebic meningoencephalitis reports.

Number	Year	Author	Age	Sex	Contact	Diagnosis	Treatment	Outcome
1	1971	Pan et al. [11]	3 y	M	Yes	Wet smear	Amphotericin B Sulphadiazine Dexamethasone	Cured
2	1971	Pan et al. [11]	5 m	M	—	Wet smear	Amphotericin B Sulphadiazine Streptomycin	Cured
3	1998	Singh et al. [12]	8 y	M	No	Wet smear	Amphotericin B Rifampin	Cured
4	2002	Shenoy et al. [13]	4 m	M	No	Wet smear	Amphotericin B Ceftriaxone	Died
5	2005	Hebbar et al. [14]	5 m	M	Yes	Wet smear	Amphotericin B Chloramphenicol metronidazole	Died
6	2011	Vinay et al. [15]	5 m	M	—	Wet smear	Amphotericin B Ceftazidim Rifampin	Died
7	present	Heydari	5 m	M	No	Wet smear	Amphotericin B Rifampin	Cured
